# Supplementary material for: TMC1 and TMC2 are cholesterol-dependent scramblases that regulate membrane homeostasis in auditory hair cells
Source: bioRxiv. 2025 Jul 4:2025.07.03.663083. Preprint. [Version 1] doi: 10.1101/2025.07.03.663083 (PMC12236488; doi:10.1101/2025.07.03.663083)
Supplement: 1 [file NIHPP2025.07.03.663083V1-supplement-1.pdf]

bioRxiv preprint doi: https://doi.org/10.1101/2025.07.03.663083; this version posted July 4, 2025. The copyright holder for this preprint (which was not certified by peer review) is the author/funder. This article is a US Government work. It is not subject to copyright under 17 USC 105 and is also made available for use under a CC0 license.

Supplemental information:

Supplementary Figures S1–S7

Supplementary Table 1

Supplementary Movies:

Movie S1 – Lipid scrambling during an all-atom MD simulation of CmTMC1. A scrambling event observed through CmTMC1 (red) in the presence of 10% cholesterol. A POPE (green) molecule translocated through the open pore during the ~ 1.7-μs long equilibrium MD simulation. Other lipids head groups are shown as white spheres. Protein is shown as a red molecular surface. Water molecules, ions, and lipid tails for non-translocating lipids are omitted for visualization purposes.

Movie S2 – Lipid scrambling during an all-atom MD simulation of MuTMC2. Two scrambling events observed through MuTMC2 in the presence of 10% cholesterol, where both POPC (gray) and POPE (green) molecules translocated through the open pore during the 1.7-μs long equilibrium MD simulation. System shown as in Movie S1 with protein in blue.

Movie S3 – Canonical lipid scrambling during a CG MD simulation of MuTMC2. A POPC lipid (1207) translocated through the open pore subunit of the CG MuTMC2 dimeric complex without cholesterol. Most translocation events are through the open pore. System shown as in Movie S2.

Movie S4 – A rare non-canonical scrambling event during a CG MD simulation of CmTMC1. A POPC lipid (807) translocated through the closed pore subunit of the CG CmTMC1 dimeric complex without cholesterol. System shown as in Movie S1.

Movie S5 – A rare non-canonical scrambling event during a CG MD simulation of MuTMC2. A POPC lipid (1174) translocated through the TM10 dimerization interface of the CG MuTMC2 dimeric complex in the presence of 10% cholesterol. System shown as in Movie S2.

Movie S6 – A rare non-canonical scrambling event during a CG MD simulation of MuTMC2. A POPS lipid (1371) translocated through the TM10 dimerization interface of the CG MuTMC2 dimeric complex in the presence of 10% cholesterol. System shown as in Movie S2.

bioRxiv preprint doi: https://doi.org/10.1101/2025.07.03.663083; this version posted July 4, 2025. The copyright holder for this preprint (which was not certified by peer review) is the author/funder. This article is a US Government work. It is not subject to copyright under 17 USC 105 and is also made available for use under a CC0 license.

SUPPLEMENTARY FIGURE 1

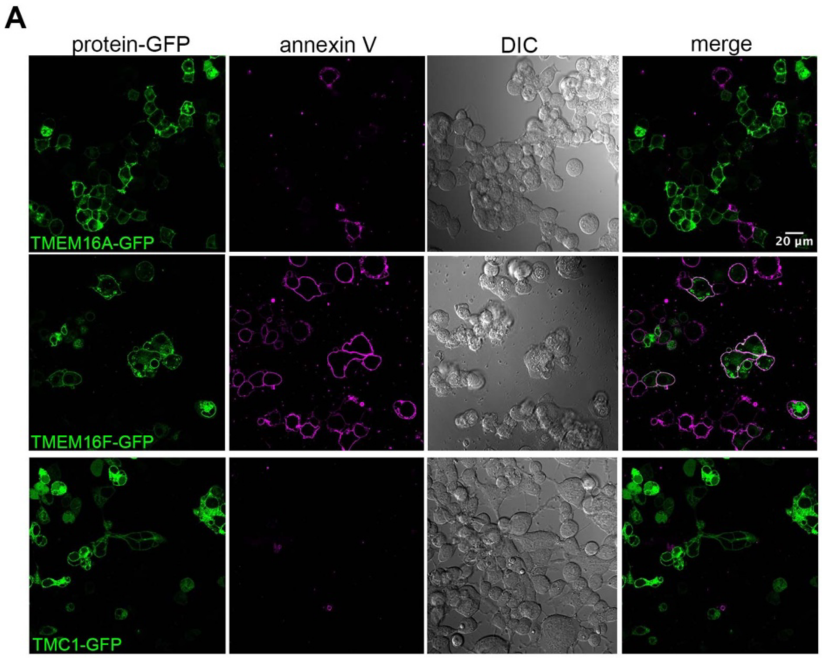

Supplementary figure 1: TMC1 does not trigger PS externalization when expressed in HEK293 cells. HEK293T cells transiently expressing the chloride channel TMEM16A or mTMC1 tagged with GFP (green) lacked externalized PS as observed by the absence of annexin V labeling (magenta). However, HEK293T cells expressing the lipid scramblase TMEM16F showed externalized PS at the plasma membrane that was detected by annexinV-647, indicating that TMEM16F function as a lipid scramblase. DIC is also included to appreciate the presence of non-transfected cells.

bioRxiv preprint doi: https://doi.org/10.1101/2025.07.03.663083; this version posted July 4, 2025. The copyright holder for this preprint (which was not certified by peer review) is the author/funder. This article is a US Government work. It is not subject to copyright under 17 USC 105 and is also made available for use under a CC0 license.

SUPPLEMENTARY FIGURE 2

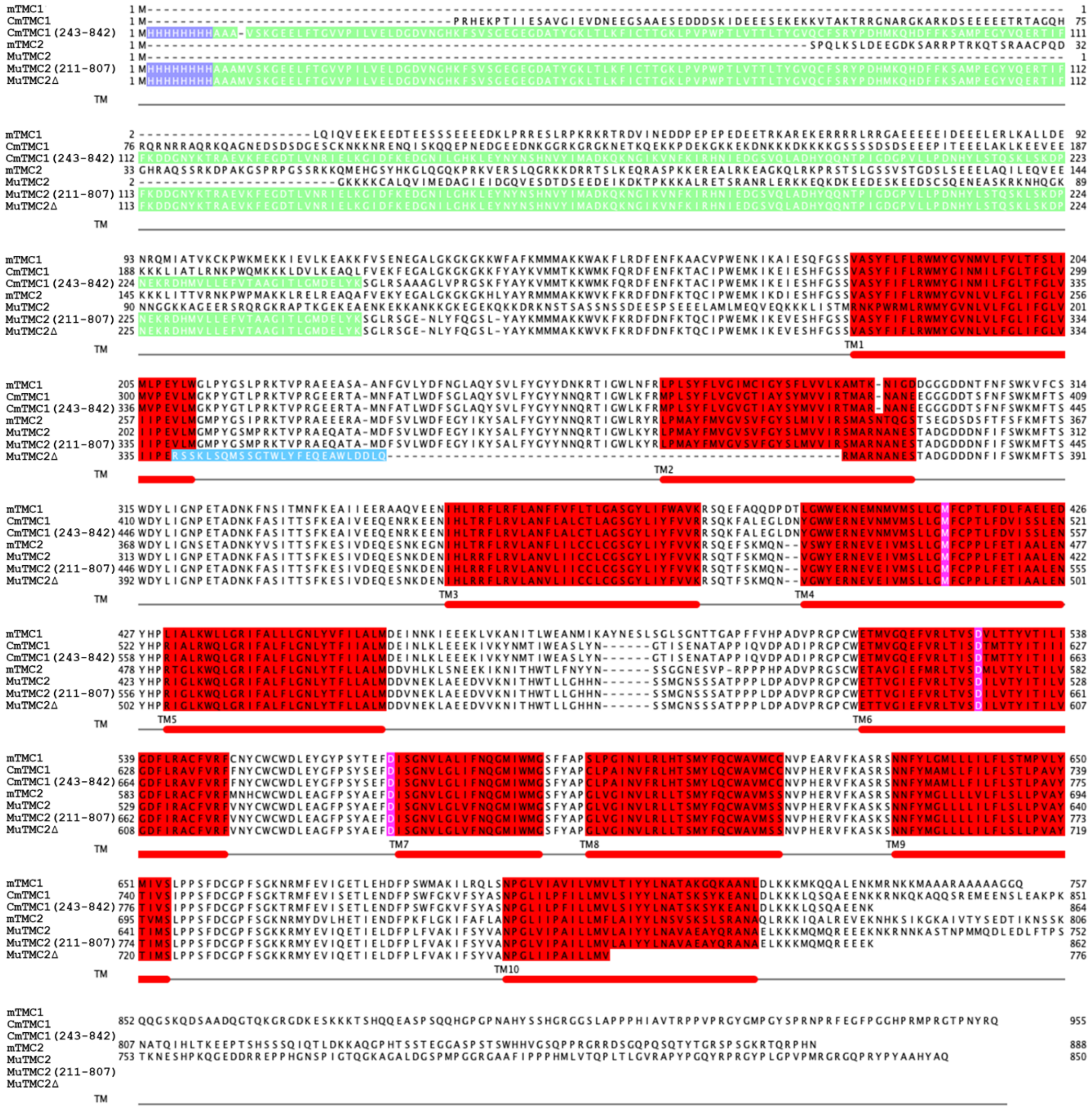

Supplementary figure 2: Sequence alignment of the TMC1/2 proteins used in this study. Sequence alignment of mTMC1, CmTMC1 full-length, CmTMC1 lacking the N- and C-terminals (243-842), mTMC1, MuTMC2 full-length, MuTMC2 lacking the N- and C-terminals (211-807), and MuTMC2Δ. Histidine tag is highlighted in purple, GFP in green, TM segments in red and the insertion present in MuTMC2Δ in blue. TM segments are also indicated below the alignment and deafness-causing mutations are shown in magenta.

bioRxiv preprint doi: https://doi.org/10.1101/2025.07.03.663083; this version posted July 4, 2025. The copyright holder for this preprint (which was not certified by peer review) is the author/funder. This article is a US Government work. It is not subject to copyright under 17 USC 105 and is also made available for use under a CC0 license.

SUPPLEMENTARY FIGURE 3

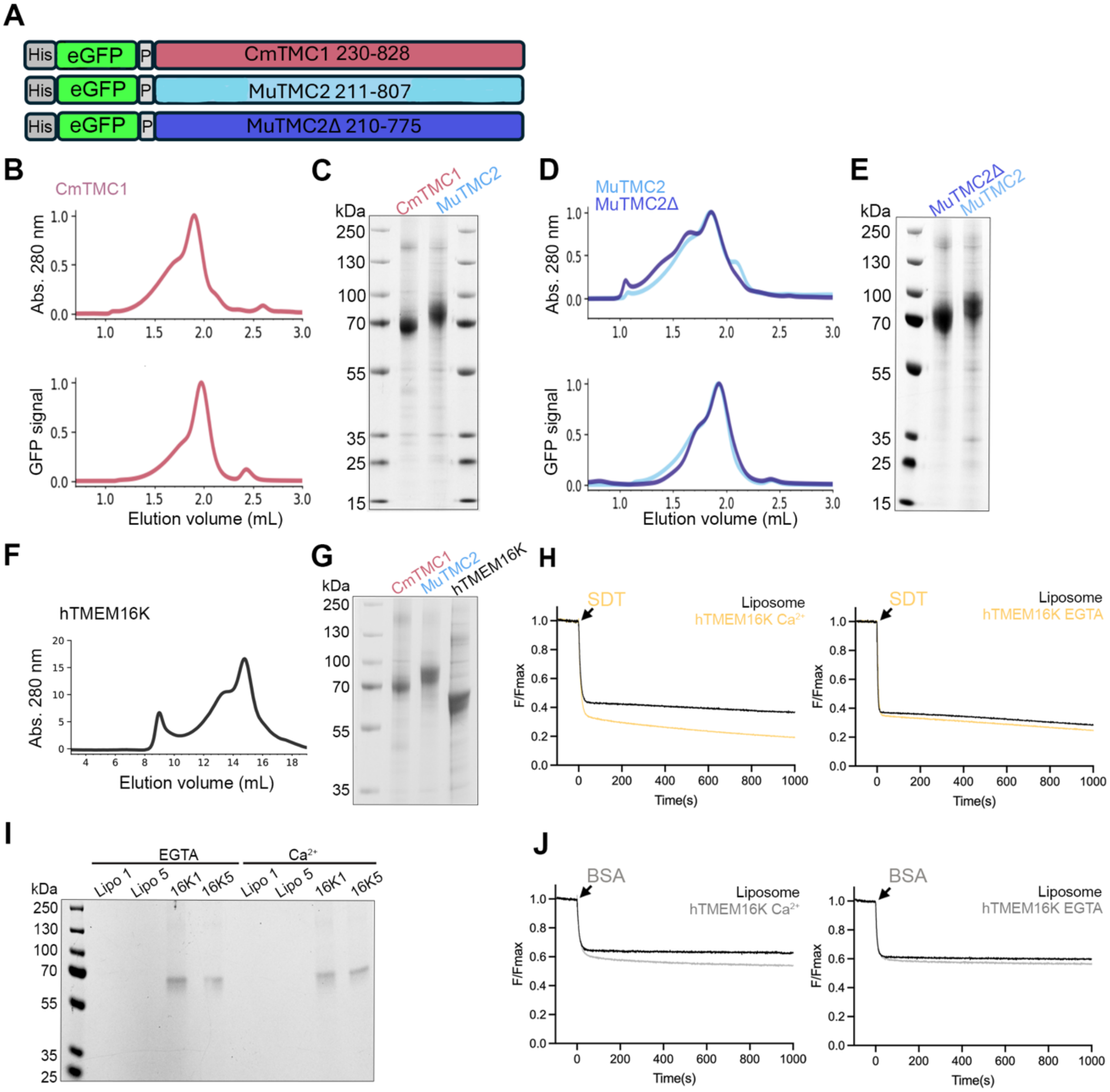

Supplementary figure 3: Protein purification and hTMEM16K scramblase assays. A. Cartoon representing the CmTMC1, MuTMC2 and MuTMC2Δ constructs used in this study. B. FSEC profiles showing the normalized absorbance at 280 nm (top panel) or GFP signal (bottom panel) of purified CmTMC1 in DDM/CHS. C. SDS-PAGE gel showing the CmTMC1 and MuTMC2 proteins used in our experiments. Protein bands and estimated molecular weights for the protein ladder are indicated. D. FSEC profiles showing the absorbance at 280 nm (top panel) and GFP signal (bottom panel) of purified MuTMC2 (light blue) and MuTMC2Δ (dark blue) in DDM/CHS. E. SDS-PAGE gel showing the MuTMC2 (light blue) and MuTMC2Δ (dark blue) proteins used in our experiments. F. FSEC profile showing the absorbance at 280 nm of purified hTMEM16K in DDM/CHS. G.SDS-PAGE gel showing purified CmTMC1, MuTMC2 and hTMEM16K used in our experiments. H-J. Fluorescence intensity decay over time due to SDT (yellow) or BSA (gray) addition to POPC/PE/PS liposomes (black trace) or hTMEM16K proteo-liposomes in the presence of 0.5 mM Ca^{2+} (left) or 2 mM EGTA (right). I. SDS- PAGE gels of representative liposome (Lipo1/5, indicating wash 1 or 5) or hTMEM16K proteo-liposome (16K1/5) samples used in our experiments, confirming the presence of protein in the proteo-liposomes.

bioRxiv preprint doi: https://doi.org/10.1101/2025.07.03.663083; this version posted July 4, 2025. The copyright holder for this preprint (which was not certified by peer review) is the author/funder. This article is a US Government work. It is not subject to copyright under 17 USC 105 and is also made available for use under a CC0 license.

SUPPLEMENTARY FIGURE 4

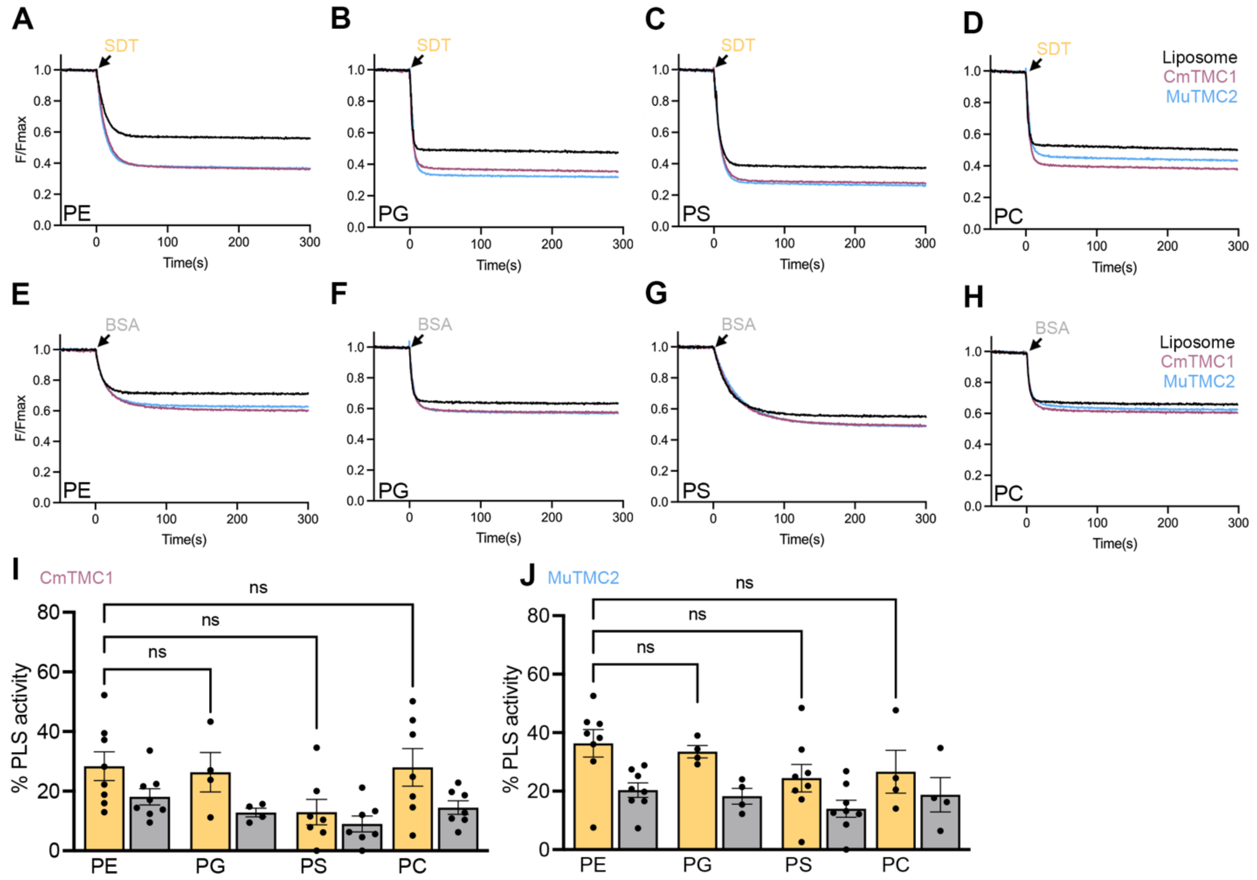

Supplementary figure 4: CmTMC1 and MuTMC2 scramble phospholipids with different head groups. A-D. Representative traces of NBD fluorescence decay after SDT addition to POPC/PE/PS/10CHO liposomes (black) containing 0.4% of acyl-NBD-labeled PE (A), PG (B), PS (C), or PC (D) or proteo-liposomes containing CmTMC1 (red) or MuTMC2 (blue). E-H. Representative traces of NBD fluorescence decay after BSA addition to the same samples from (A-D). I. Quantification of CmTMC1 SDT- (yellow) and BSA (grey) -based scramblase activity in the conditions shown in A-H. J. Quantification of MuTMC2 SDT- (yellow) and BSA (grey) -based scramblase activity in the conditions shown in A-H. Each dot represents one independent experiment. Mean ± SEM is shown for 3-7 independent experiments. One-way ANOVA was performed to evaluate statistical significance between NBD-PE and the other NBD-lipids. K. SDS-PAGE gels of representative liposome (Lipo1-5) or TMC proteo-liposomes (W1-5) samples used in our experiments, confirming the presence of protein in the proteo-liposomes samples.

bioRxiv preprint doi: https://doi.org/10.1101/2025.07.03.663083; this version posted July 4, 2025. The copyright holder for this preprint (which was not certified by peer review) is the author/funder. This article is a US Government work. It is not subject to copyright under 17 USC 105 and is also made available for use under a CC0 license.

SUPPLEMENTARY FIGURE 5

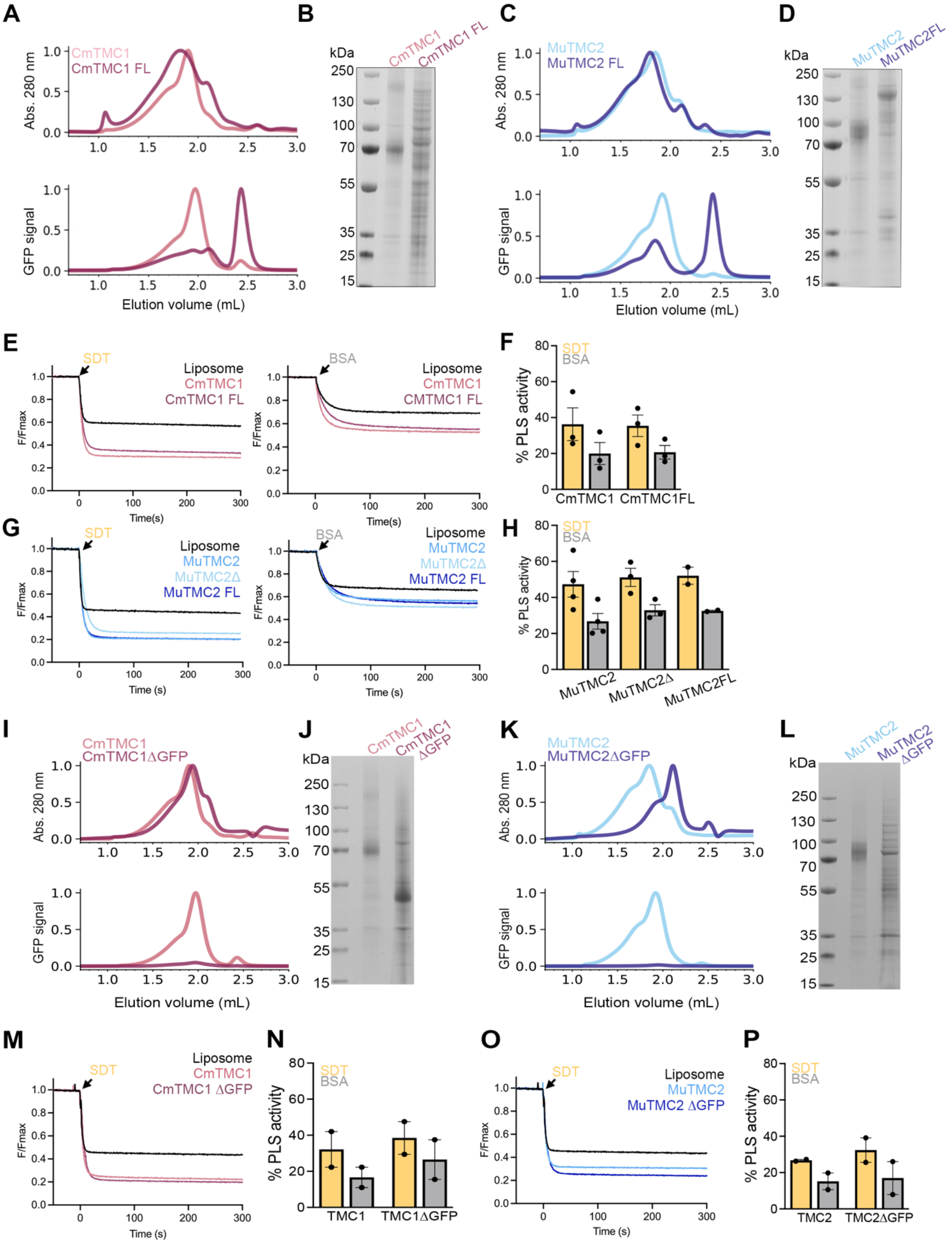

bioRxiv preprint doi: https://doi.org/10.1101/2025.07.03.663083; this version posted July 4, 2025. The copyright holder for this preprint (which was not certified by peer review) is the author/funder. This article is a US Government work. It is not subject to copyright under 17 USC 105 and is also made available for use under a CC0 license.

Supplementary figure 5: TMC scramblase activity resides at the TMC core. A. FSEC profiles showing the normalized absorbance at 280 nm (top) or GFP signal (bottom) of purified truncated (light red) or full-length (FL, dark red) CmTMC1. B. SDS-PAGE gel of the proteins shown in (A). Protein bands and estimated molecular weights for the protein ladder are indicated. C. FSEC profiles showing the normalized absorbance at 280 nm (top) or GFP signal (bottom) of purified truncated (light blue) or FL MuTMC2 (dark blue). D. SDS- PAGE gel of the proteins shown in (C). E. Fluorescence intensity decay over time due to SDT (left) or BSA (right) addition to POPC/PE/PS/10CHO liposomes (black trace) or proteo-liposomes containing truncated (light red) or full-length (dark red) CmTMC1. F. Quantification of the SDT- (yellow) or BSA- (grey) scramblase activity of truncated or FL CmTMC1. G. Fluorescence intensity decay over time due to SDT (left) or BSA (right) addition to POPC/PE/PS/10CHO liposomes (black trace) or proteo-liposomes containing truncated truncated (MuTMC2), previously published MuTMC2Δ, or MuTMC2 FL. H. Quantification of the SDT- (yellow) or BSA- (grey) scramblase activity of the MuTMc2 constructs shown represented in G. Mean ± SEM is shown. Each dot represents one independent experiment. One-way ANOVA was performed to evaluate statistical significance between the SDT activity of the TMC proteo-liposomes, but no statistical significance was found in F or H. I. FSEC profiles showing the normalized absorbance at 280 nm (top) and GFP signal (bottom) of CmTMC1(light red) or tagless CmTMC1 (ΔGFP, dark red). J. SDS- PAGE gel of the proteins shown in (I). K. FSEC profiles showing the normalized absorbance at 280 nm (top) and GFP signal (bottom) of MuTMC2 (light blue) or tagless MuTMC2 (ΔGFP, dark blue). L. SDS- PAGE gel of the proteins shown in (K). M. Fluorescence intensity decay over time due to SDT addition to POPC/PE/PS/10CHO liposomes (black) or proteo-liposomes containing CmTMC1 (light red) or CmTMC1 ΔGFP (dark red). N. Quantification of the scramblase activity of CmTMC1 or CmTMC1 ΔGFP. O. Fluorescence intensity decay over time due to SDT addition to POPC/PE/PS/10CHO liposomes (black) or proteo-liposomes containing MuTMC2 or MuTMC2 ΔGFP. P. Quantification of the scramblase activity of MuTMC2 or MuTMC2 ΔGFP. Mean ± SEM is shown. Each dot represents one independent experiment. One-way ANOVA was performed to evaluate statistical significance between the SDT activity of the TMC proteo-liposomes, but no statistical significance was

bioRxiv preprint doi: https://doi.org/10.1101/2025.07.03.663083; this version posted July 4, 2025. The copyright holder for this preprint (which was not certified by peer review) is the author/funder. This article is a US Government work. It is not subject to copyright under 17 USC 105 and is also made available for use under a CC0 license.

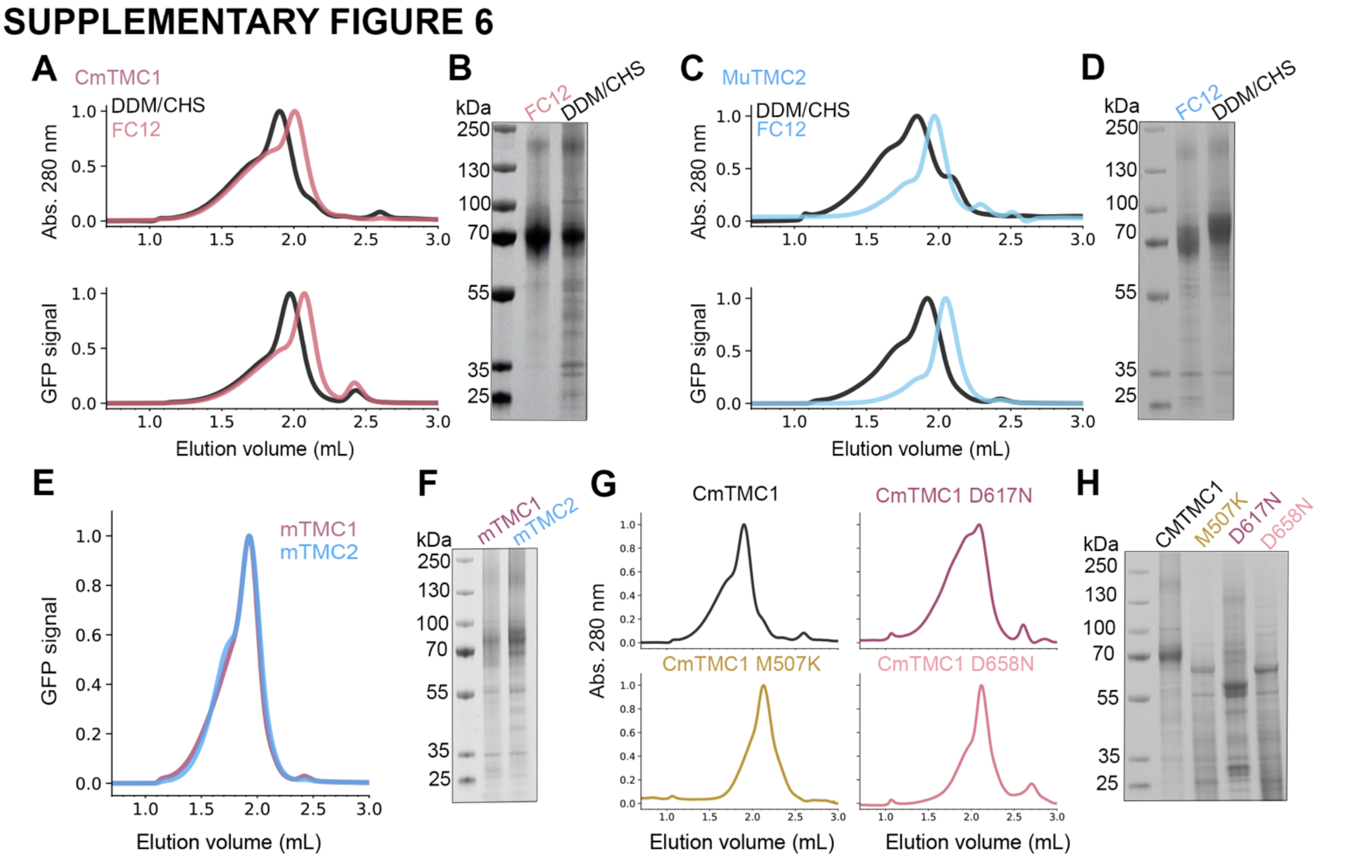

Supplementary figure 6: Purification of CMTMC1 and MuTMC2, mouse TMC1 and TMC2 and CmTMC1 deafness-causing mutations. A. FSEC profiles showing the normalized absorbance at 280 nm (top) and GFP signal (bottom) of purified CmTMC1 in FC12 (pink) or DDM/CHS (black). B. SDS- PAGE gel of the CmTMC1 proteins shown in (A). Protein bands and estimated molecular weights for the protein ladder are indicated. C. FSEC profiles showing the normalized absorbance at 280 nm (top) and GFP signal (bottom) of purified MuTMC2 in FC12 (blue) or DDM/CHS (black). D. SDS- PAGE gel of the MuTMC2 proteins shown in (C). E. FSEC profiles showing the normalized absorbance at 280 nm (top) of purified mTMC1 (pink) and mTMC2 (blue) used in our experiments. F. SDS- PAGE gel of the proteins shown in (E). G. Fluorescence intensity decay over time due to BSA addition to POPC/PE/PS liposomes (black) without (right) or with 10% cholesterol (left) or proteo-liposomes containing mTMC1 (red) or mTMC2 (blue). H. FSEC profiles showing the normalized absorbance at 280 nm (top) of purified CmTMC1 mutants used in our experiments. I. SDS-PAGE gel of the proteins shown in (H).

bioRxiv preprint doi: https://doi.org/10.1101/2025.07.03.663083; this version posted July 4, 2025. The copyright holder for this preprint (which was not certified by peer review) is the author/funder. This article is a US Government work. It is not subject to copyright under 17 USC 105 and is also made available for use under a CC0 license.

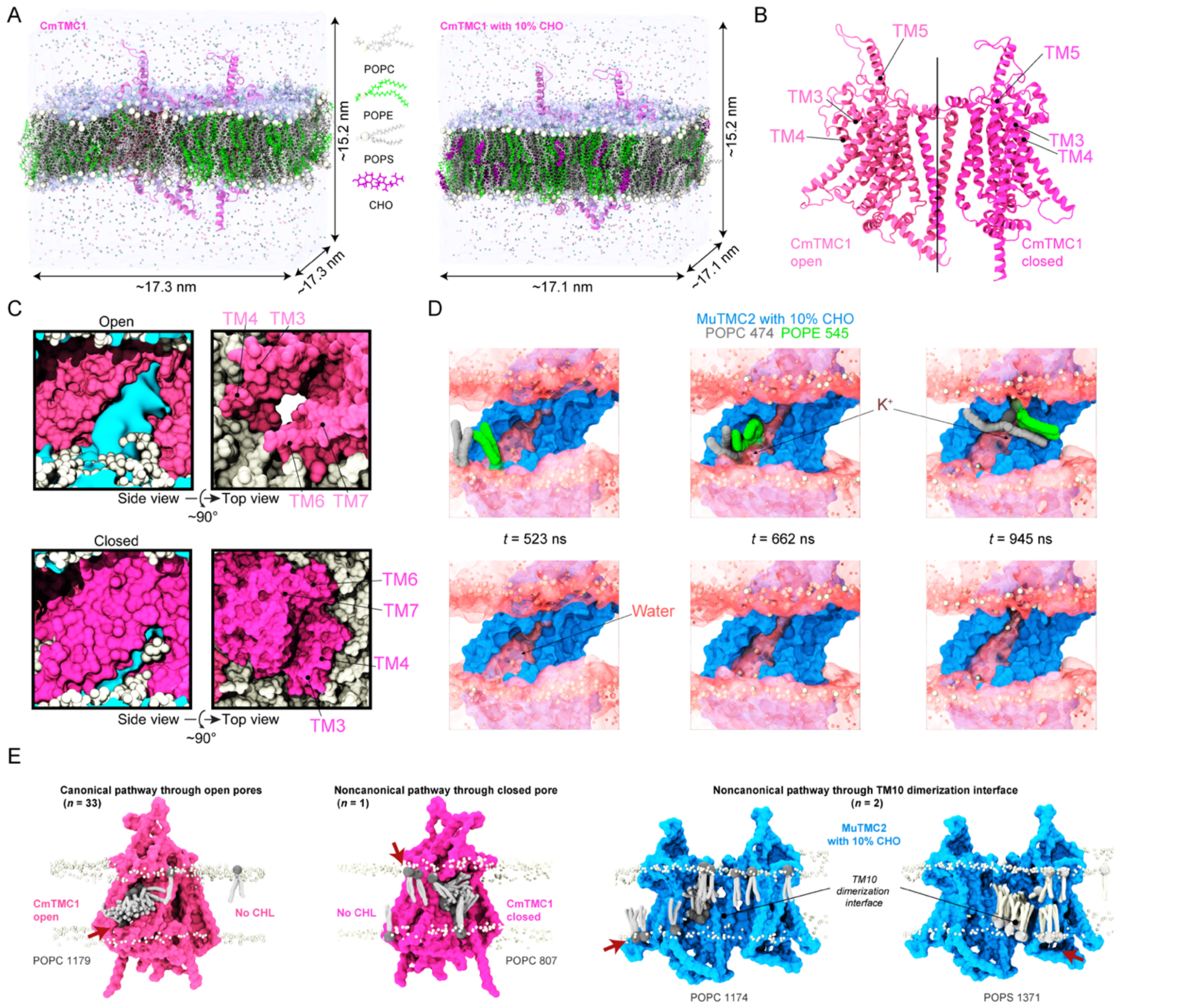

Supplemental Figure 7: MD simulations reveal lipid scrambling by CmTMC1 and MuTMC2 through open-like groove. A. AlphaFold3-predicted dimeric model of CmTMC1 embedded into a mixed lipid POPC/PE/PS bilayers without (left) and with (right) 10% cholesterol. Similar systems were built for MuTMC2. Proteins are shown in ribbon representation, lipid phosphate atoms are shown as white spheres, water is transparent, and ions are shown as spheres. Lipids are shown as indicated in inset. B. Illustration of CmTMC1 after TMD-induced opening of one pore. C. Side and top views of one subunit of CmTMC1 where TMD simulations were used to open the pore region (top) or with a closed pore (bottom). Protein is shown in molecular surface representation, lipid headgroups are shown as white spheres, and water is in cyan. D. Snapshots at indicated time points during the lipid translocation events in the all-atom MuTMC2/10CHO system. Potassium ions (dark grey) and water molecules (red) occupied the open groove as the lipids translocated. E. Canonical (left) and rare noncanonical scrambling events (three right panels), including a scrambling event where POPC 807 translocates through the closed pore CmTMC1 and two other events (POPC 1174 and POPS 1371) showing scrambling through the TM10 dimerization interface in MuTMC2 with 10% cholesterol. In each case a translocating single lipid is shown at different time points overlaid on a representative structural model of the protein. Non-translocating lipid phosphate beads are shown as white spheres. Other system components are omitted for visualization purposes.

bioRxiv preprint doi: https://doi.org/10.1101/2025.07.03.663083; this version posted July 4, 2025. The copyright holder for this preprint (which was not certified by peer review) is the author/funder. This article is a US Government work. It is not subject to copyright under 17 USC 105 and is also made available for use under a CC0 license.

Supplementary Table 1: Summary of simulations and scrambling events.

Label	System	t_{sim} (ns)	Type	Start	Size (#atoms/ beads)	Initial Size (nm^3)	Number of Scrambling events	Scrambling rates ($lipids \cdot s^{-1}$)
S1a	CmTMC1 POPC / POPE / POPS	100	EQ	-	408,861	17×17×15	0	0
S1b		20	TMD	S1a			0	0
S1c		1,500	TMD-CNST	S1b			0	0
S1d		10,000	CG-EQ	S1b	68,366	18×18×22	3	0.3×10^6
S2a	CmTMC1 POPC / POPE / POPS / 10CHO	100	EQ	-	403,998	17×17×15	0	0
S2b		20	TMD	S2a			0	0
S2c		1,700	TMD-CNST	S2b			1	0.6×10^6
S2d		10,000	CG-EQ	S2b	68,211	18×18×22	11	1.1×10^6
S3a	MuTMC2 POPC / POPE / POPS	100	EQ	-	413,104	17×17×15	0	0
S3b		20	TMD	S3a			0	0
S3c		1,500	TMD-CNST	S3b			0	0
S3d		10,000	CG-EQ	S3b	68,391	18×18×22	6	0.6×10^6
S4a	MuTMC2 POPC / POPE / POPS / 10CHO	100	EQ	-	405,586	17×17×15	0	0
S4b		20	TMD	S4a			0	0
S4c		1,700	TMD-CNST	S4b			2	1.2×10^6
S4d		10,000	CG-EQ	S4b	68,030	18×18×22	16	1.6×10^6
